# Factors influencing vehicle passenger fatality have changed over 10 years: a nationwide hospital-based study

**DOI:** 10.1038/s41598-020-60222-z

**Published:** 2020-02-24

**Authors:** Wataru Ishii, Masahito Hitosugi, Arisa Takeda, Mineko Baba, Ryoji Iizuka

**Affiliations:** 1 Kyoto Daini Red Cross Hospital, Critical Care Center, Emergency of Medicine, Haruobi, Kamazamarutamachi, Kamigyo, Kyoto, 602-8026 Japan; 2 Department of Legal Medicine, Shiga University of Medical Science, Tsukinowa, Seta, Otsu, Shiga, 520-2192 Japan; 3 Center for Integrated Medical Research, Keio University School of Medicine, Tokyo, Japan

**Keywords:** Health care, Health care, Outcomes research, Outcomes research

## Abstract

Traffic injury trends have changed with safety developments. To establish effective preventive measures against traffic fatalities, the factors influencing fatalities must be understood. The present study evaluated data from a national medical database to determine the changes in these factors over time, as this has not been previously investigated. This observational study retrospectively analysed data from the Japanese Trauma Data Bank. Vehicle passengers involved in collisions from 2004–2008 and 2016–2017 were included. Data were compared between the two study periods, and between fatal and non-fatal patients within each period. Multivariate logistic regression analyses were performed to determine the factors influencing fatalities. In 2016–2017, patients were older and had lower fatality rates. In 2004–2008, fatalities were more likely to involve older male front-seat passengers with low d-BP, BT, and GCS values, and high AIS of the neck and abdomen. However, in 2016–2017, fatalities were more likely to involve older males with low GCS, high AIS of the abdomen, and positive focused assessment with sonography for trauma results. Our study identified independent factors influencing vehicle passenger fatalities, which will likely continue to evolve with the aging of the population and changing manners of injury.

## Introduction

A motor vehicle collision (MVC) is defined as a collision involving at least one moving vehicle that occurred or originated on a road or street open to public traffic and resulted in one or more persons being killed or injured^1^. Worldwide, the number of fatalities from MVCs in 2004 was higher than those from malaria (ranked 9th and 13th, respectively)^2^. The number of MVCs is continuously increasing, and MVC was the cause of 1.35 million fatalities in 2016^3^. MVCs are predicted to become the 5th most common cause of fatalities in the world by 2030^4^. Therefore, it is recommended that all countries make greater efforts to decrease MVC fatalities. The value of setting targets to improve road safety performance was acknowledged in the Paris-based Organisation for Economic Co-operation and Development (OECD) report, Safety on the Road, in 2002^5^. Target-setting is recommended for all countries attempting to reduce road fatalities.

Technological advancements and road improvements have been made to prevent MVCs and decrease the severity of injuries incurred by vehicle occupants; improvements in vehicle crashworthiness have increased the collision survivability of motor vehicle passengers, and the focus of recent automobile designs is to avoid crashes altogether^6^. Since the 1990s, vehicle safety technologies such as airbags and electronic stability control have been developed. As a result, the MVC mortality decreased to its lowest level of 1.08 fatalities per 100 million vehicle miles travelled in 2014^7^. Recent crash avoidance technologies, such as side view assist, forward collision warning/mitigation, lane departure warning/prevention, and adaptive headlights have great potential for preventing crashes^6^. The motor vehicle fatality rate may be further reduced by improved road conditions, clear zone sizes and clearing of roadside vegetation, improved driver behaviour, and modified speed limits during inclement weather^8,9^.

The Japanese government established the Traffic Safety Measures Basic Law in 1970^10^. With this law, the Traffic Safety Basic Plan specified goals every 5 years after 1971, and traffic safety measures were promoted comprehensively and systemically. The essential goal is to achieve a society with no traffic accidents. For the period from 2011–2015, the target was fewer than 3,000 fatalities and 700,000 casualties^11^. However, although the goal for casualties was achieved by the end of 2015 (670,140), the goal for fatalities was not (4,117)^11^. The Japanese government established a new set of objectives in 2016 to reduce the number of fatalities to fewer than 2,500 and the number of casualties to fewer than 500,000 by 2020^11^. These goals necessitate the continuous elimination of traffic fatalities in Japan. Data from 2017 show that most motor vehicle injuries in Japan are incurred by motor vehicle drivers (64.1%), followed by bicyclists (15.9%), motorcyclists (10.3%) and pedestrians (9.5%)^12^. An evaluation of the factors that led to these injuries would be useful in establishing effective measures to prevent motor vehicle fatalities.

Many studies have investigated the circumstances under which motor vehicle passengers are more likely to be killed or severely injured in MVCs, and have evaluated the factors affecting the severity of MVC injuries^13–16^. However, these studies were based on police data or crash databases that contained information regarding the type of vehicle involved, situation of the collision, major injuries incurred by the victims, and other associated factors. No studies have examined the factors influencing the occurrence of motor vehicle passenger fatalities based on nationwide medical data obtained from hospitals. The main objective of the present study was to examine the data from a nationwide trauma database to identify the factors influencing MVC fatalities; this information will aid in the establishment of effective preventive measures. In addition, as traffic injury trends have changed with traffic safety development, the present study investigated the changing factors influencing fatalities.

## Methods

### Study design and patient selection

This observational study was a retrospective analysis of the data from a national hospital-based database, the Japanese Trauma Data Bank (JTDB). The JTDB is a nationwide trauma registry in Japan that contains data recorded since 2003 by the Japanese Association for the Surgery of Trauma^17^. Approximately 294,000 trauma patients were registered until 2017^18^. Fifty-five hospitals participated in the registry in 2005, and over 272 hospitals participated in the registry in 2017; this accounted for approximately 75% of the critical care centres in Japan. The inclusion criteria for the JTDB were patients with trauma who had an Abbreviated Injury Scale (AIS) of 3 or more. The JTDB collected information on the manners and mechanisms of injuries, vital signs, anatomical and physiological injury severity, pre-hospital and in-hospital treatment, and outcome.

**Figure 1:**
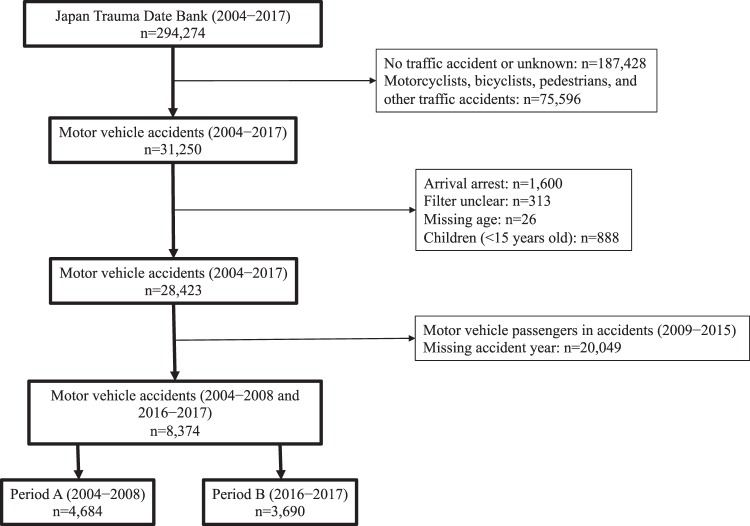
Flowchart of patient enrolment.

Date were obtained from the JTDB in December 2018. A total of 294,274 patients were registered in the JTDB from 2004–2017. Of these, 31,250 were MVC-related trauma patients. Cases were excluded if the patient arrived with cardiopulmonary arrest (n = 1,600), if they had unclear or missing data (n = 339), and if they were younger than 15 years old (n = 888). The data from 28,423 vehicle passengers (including drivers) were assessed. Among them, the cases that occurred from 2004–2008 (Period A) and 2016–2017 (Period B) were selected. Period A included data from 4,684 patients, while Period B included data from 3,690 patients (Fig. 1). As only a small number of hospitals participated in the registry in its early period, Period A comprised 5 years of data, while Period B comprised 2 years of data.

### Collected data

The JTDB was created in 2003 by the Japanese Association for the Surgery and Trauma (Trauma Surgery Committee) and the Japanese Association for Acute Medicine (Committee for Clinical Care Evaluation^19,20^), similar to trauma databases in North America, Europe, and Oceania^21^. From the JTDB database, the following information was obtained for each patient: age, sex, vehicle seating position, duration between the emergency call and hospital arrival, vital signs on arrival to the hospital including systolic and diastolic blood pressure (s-BP and d-BP), heart rate (HR), respiration rate (RR), body temperature (BT), Glasgow coma score (GCS), focused assessment with sonography for trauma (FAST) examination results, AIS score (version 1998) for each body region, Injury Severity Score (ISS), and hospital discharge outcome. The AIS score is used to categorise the injury type and severity anatomically in each body region on a scale from 1 (minor) to 6 (clinically untreatable). The ISS, which is useful for assessing the severity of multiple injuries, is the sum of the squares of the highest AIS score in each of the three most severely injured body regions.

### Statistical analysis

Categorial variables were summarised in the form of values with proportions or frequencies. Continuous variables were summarised mean ± standard deviation for the values that followed the normal distribution, and as the median and interquartile range (IQR) for values that were not normally distributed. Chi-square tests were used to compare prevalence between the two groups. To find the differences in values between two groups, the Student’s t-test was used for values with normal distribution, while the Mann-Whitney test was conducted for values without normal distribution. A P value of 0.05 or less was considered statistically significant. To identify which variables were independently associated with poor outcome, a logistic regression analysis was performed. Risk factors with P values <0.05 in univariable analysis were included in the multivariable model. The analyses were performed with SPSS Ver. 23.

### Endpoint

The changes in factors influencing traffic fatalities were examined using the data from the JTDB.

### Ethics

Personal identifiers had already been removed from the JTDB. This study was conducted in accordance with the Declaration of Helsinki, and was approved by the Ethical Committee of Kyoto Daini Red Cross Hospital (Sp 2019–10). Because of the anonymous and retrospective nature of this study, the need for informed consent was waived by the Ethics Committee that approved the study protocol.

## Results

### Comparisons between the two periods

Patient backgrounds characteristics and vital signs at the time of hospital arrival were compared between Period A (2004–2008) and Period B (2016–2017). A marked aging trend was observed in Japan between these two periods. In 2006, the average life span was 79.0 years for males and 85.8 years for females, and people aged 65 years or older accounted for 20.8% of the total population^22^. In 2016, the average life spans had increased to 81.0 years for males and 87.1 years for females, with people aged 65 years or older comprising 27.3% of the population^23^. Compared with Period A, the patients in Period B had a significantly older average age and a significantly greater proportion of females (Table 1). The proportion of front-seat passengers was significantly lower in Period B than Period A, while the proportion of rear-seat passengers was significantly higher in Period B than Period A. The duration between the emergency call and hospital arrival was significantly shorter in Period B than in Period A. On arrival at the hospital, the average s-BP, d-BP, BT, and GCS values were significantly higher in Period B than in Period A. In contrast, the HR, RR, and proportion of FAST positivity were significantly lower in Period B than in Period A. There was also significantly lower mortality in Period B than in Period A.

**Table 1:** Patient characteristics and vital signs at the time of hospital arrivals in Period A versus Period B.

	Period A (2004 to 2008)	Period B (2016 to 2017)	P value
	(n = 4,684)	(n = 3,690)	
Age (years)	45.1 ± 19.2	52.7 ± 21.4	<0.001
Sex, n (%)			<0.001
Male	69.0	61.6	
Female	31.0	38.4	
Seating position (%)			0.038
Driver	77.4	76.8	
Front seat passenger	14.4	13.5	
Rear seat passenger	8.2	9.6	
Duration between emergency call to hospital arrival(min)	55 ± 46	48 ± 39	<0.001
Systolic blood pressure (mmHg)	129.7 ± 32.4	134.9 ± 31.5	<0.001
Diastolic blood pressure (mmHg)	77.1 ± 18.9	80.9 ± 19.9	<0.001
Heart rate (beats/min)	88.4 ± 21.1	86.2 ± 18.8	<0.001
Respiration rate (breaths/min)	22.8 ± 7.3	21.5 ± 6.7	<0.001
Body temperature (°C)	36.3 ± 0.9	36.6 ± 0.8	<0.001
Glasgow coma scale	13.5 ± 3.0	13.9 ± 2.5	<0.001
FAST positive (%)	15.2	11.6	<0.001
Death (%)	7.9	4.3	<0.001

FAST: focused assessment with sonography for trauma.

Injury severity was then compared between the two periods. There was no significant difference in the ISS of the patients between the two periods (Table 2). The highest AIS score was attained for the chest, followed by the head and lower extremities in both periods. The AIS scores of the head, face, abdomen, and lower extremities were significantly lower in Period B than in Period A. In contrast, the AIS scores of the chest and spine were significantly higher in Period B than in Period A.

**Table 2:** Injury Severity Score (ISS) and Abbreviated Injury Scale (AIS) by body region in Period A versus Period B.

	Period A (2004 to 2008)	Period B (2016 to 2017)	P value
	(n = 4,684)	(n = 3,690)	
ISS, median (IQR)	13.0 (8.0–21.0)	13.0 (9.0–20.0)	0.595
AIS, median (IQR)			
Head*	0.0 (0.0–2.0)	0.0 (0.0–2.0)	0.007
Face*	0.0 (0.0–1.0)	0.0 (0.0–0.0)	<0.001
Neck	0.0 (0.0–0.0)	0.0 (0.0–0.0)	0.612
Chest**	0.0 (0.0–3.0)	1.0 (0.0–3.0)	<0.001
Abdomen*	0.0 (0.0–1.0)	0.0 (0.0–0.0)	0.002
Spine**	0.0 (0.0–0.0)	0.0 (0.0–1.0)	<0.001
Upper extremities	0.0 (0.0–1.0)	0.0 (0.0–0.0)	0.081
Lower extremities*	0.0 (0.0–2.0)	0.0 (0.0–1.0)	<0.001

*Median values were significantly higher in Period A than Period B.

**Median values were significantly lower in Period A than Period B.

### Comparisons of patient outcome during Period A

Of the patients in Period A, 301 (7.9%) died (fatal group) and 3,527 survived (non-fatal group). Patient background characteristics and vital signs at the time of hospital arrival were compared between the fatal and non-fatal groups. Compared with the non-fatal group, the fatal group had a significantly higher mean age and proportion of males, and a significantly lower proportion of front-seat passengers (Table 3). At the hospital, the d-BP, BT, and GCS were significantly lower in the fatal group than in the non-fatal group. Regarding injury severity, the ISS and AIS of the neck and abdomen were significantly higher in the fatal group than in the non-fatal group (Table 4).

**Table 3:** Patient characteristics and vital signs at the time of hospital arrival of patients who survived (non-fatal group) versus patients who died (fatal group) during Period A (2004–2008).

	Non-fatal	Fatal	OR	Lower 95% CI	Upper 95% CI	P value
	(n = 3,527)	(n = 301)				
Age (years)	44.5 ± 19.0	53.3 ± 20.9	1.036	1.024	1.047	<0.001
Sex (%)						
Male	70.2	76.1	1.948	1.143	3.321	0.014
Female	29.8	23.9	Ref			Ref
Seating position (%)						
Driver	77.6	78.4	Ref.			Ref.
Front seat passenger	14.5	12.0	1.985	1.089	3.62	0.025
Rear seat passenger	7.9	9.6	1.021	0.424	2.458	0.963
Duration between emergency call to hospital arrival(min)	48 ± 32	53 ± 93	1.002	1.000	1.004	0.073
Systolic blood pressure (mmHg)	131.7 ± 29.4	95.2 ± 46.8	1.008	0.999	1.018	0.088
Diastolic blood pressure (mmHg)	77.6 ± 18.4	64.7 ± 23.9	0.976	0.960	0.991	0.002
Heart rate (beats/min)	87.5 ± 19.9	101.7 ± 31.4	1.003	0.994	1.012	0.534
Respiration rate (breaths/min)	22.7 ± 7.0	25.5 ± 11.3	1.025	0.999	1.052	0.064
Body temperature (°C)	36.4 ± 0.8	35.6 ± 1.2	0.539	0.426	0.681	0.001
Glasgow coma scale	13.9 ± 2.4	8.6 ± 4.7	0.740	0.696	0.788	0.001
FAST positive (%)	13.7	43.6	1.266	0.713	2.247	0.421

OR: odds ratio, 95% CI: 95% confidence interval, FAST: focused assessment with sonography for trauma.

**Table 4:** Injury Severity Score (ISS) and Abbreviated Injury Scale (AIS) by body region of patients who survived (non-fatal group) versus patients who died (fatal group) during Period A (2004–2008).

	Non-fatal	Fatal	OR	Lower 95% CI	Lower 95% CI	P value
	(n = 3,527)	(n = 301)				
ISS, median(IQR)*	13 (9.0–20.0)	29 (20.8–41.0)	1.099	1.087	1.110	<0.0001
**AIS, median** (**IQR)**						
Head	0 (0.0–2.0)	0 (0.0–4.0)	1.080	0.941	1.240	0.274
Face	0 (0.0–1.0)	0 (0.0–0.0)	0.983	0.757	1.276	0.898
Neck*	0 (0.0–0.0)	0 (0.0–0.0)	1.850	1.010	3.389	0.046
Chest	0 (0.0–3.0)	2.5 (0.0–4.0)	0.961	0.852	1.085	0.520
Abdomen*	0 (0.0–1.0)	0 (0.0–3.0)	1.464	1.239	1.731	<0.001
Spine	0 (0.0–0.0)	0 (0.0–0.0)	0.849	0.698	1.033	0.102
Upper extremities	0 (0.0–1.0)	0 (0.0–0.0)	0.819	0.634	1.059	0.129
Lower extremities	0 (0.0–2.0)	0 (0.0–3.0)	1.003	0.855	1.177	0.967

OR: odds ratio, 95% CI: 95% confidence interval, IQR: interquartile range.

*Median values were significantly higher in the fatal group than the non-fatal group.

### Comparisons of patient outcome during Period B

Of the patients in Period B, 145 (4.2%) died and 3,295 survived. Patient background characteristics and vital signs at the time of hospital arrival were compared between the fatal and non-fatal groups. The mean age and proportion of males were significantly higher in the fatal group than the non-fatal group (Table 5). At the hospital, the fatal group had a significantly lower GCS and a significantly higher FAST positive rate than the non-fatal group. Regarding injury severity, the ISS and AIS of the chest, abdomen, and lower extremities were significantly higher in the fatal group than in the non-fatal group (Table 6).

**Table 5:** Patient characteristics and vital signs at the time of hospital arrival of patients who survived (non-fatal group) versus patients who died (fatal group) during Period B (2016–2017).

	Non-fatal	Fatal	OR	Lower 95% CI	Upper 95% CI	P value
	(n = 3,295)	(n = 145)				
Age (years)	52.2 ± 21.2	63.9 ± 22.1	1.050	1.033	1.068	<0.001
Sex (%)						
Male	61.3	69.0	2.493	1.236	5.031	0.011
Female	38.7	31.0	Ref.			Ref.
Seating position (%)						
Driver	76.8	74.5	Ref.			Ref.
Front seat passenger	13.7	13.8	1.762	0.773	4.016	0.178
Rear seat passenger	9.5	11.7	1.854	0.744	4.620	0.185
Duration between emergency call to hospital arrival(min)	55 ± 47	57 ± 50	1.001	0.998	1.001	0.645
Systolic blood pressure (mmHg)	136.0 ± 29.5	102.1 ± 51.2	0.995	0.981	1.009	0.5
Diastolic blood pressure (mmHg)	81.4 ± 19.1	67.0 ± 30.1	1.003	0.983	1.023	0.771
Heart rate (beats/min)	85.5 ± 18.2	99.9 ± 27.3	1.008	0.994	1.022	0.244
Respiration rate (breaths/min)	21.5 ± 6.5	21.8 ± 11.2	1.032	0.991	1.073	0.991
Body temperature (°C)	36.6 ± 0.8	36.0 ± 1.0	0.743	0.538	1.026	0.072
Glasgow coma scale	14.1 ± 2.1	8.4 ± 4.9	0.768	0.707	0.834	<0.001
FAST positive (%)	10.4	44.6	2.395	1.204	4.765	0.013

OR: odds ratio, 95% CI: 95% confidence interval, FAST: focused assessment with sonography for trauma.

**Table 6:** Injury Severity Score (ISS) and Abbreviated Injury Scale (AIS) by body region of patients who survived (non-fatal group) versus patients who died (fatal group) during Period B (2016–2017).

	Non-fatal	Fatal	OR	Lower 95% CI	Upper 95% CI	P value
	(n = 3,295)	(n = 145)				
ISS, median (IQR)*	12.0 (8.0–19.0)	27.5 (18.0–43.0)	1.102	1.087	1.116	<0.0001
AIS, median (IQR)						
Head	0.0 (0.0–2.0)	0.0 (0.0–4.0)	1.103	0.924	1.318	0.278
Face	0.0 (0.0–0.0)	0.0 (0.0–0.0)	0.750	0.444	1.270	0.284
Neck	0.0 (0.0–0.0)	0.0 (0.0–0.0)	1.019	0.504	2.063	0.958
Chest*	1.0 (0.0–3.0)	3.0(0.0–4.0)	1.282	1.080	1.523	0.005
Abdomen*	0.0 (0.0–0.0)	1.0 (0.0–3.0)	1.401	1.145	1.713	0.001
Spine	0.0(0.0–1.0)	0.0 (0.0–0.0)	0.862	0.674	1.102	0.236
Upper extremities	0.0 (0.0–0.0)	0.0 (0.0–0.0)	0.873	0.604	1.262	0.470
Lower extremities*	0.0 (0.0–1.0)	0.0 (0.0–2.0)	1.266	1.025	1.564	0.029

OR: odds ratio, 95% CI: 95% confidence interval.

*Median values were significantly higher in the fatal group than the non-fatal group.

### Factors influencing the outcome

To identify variables that were independently associated with fatality, logistic regression analyses were performed based on the univariate results. In Period A, significant differences were found in univariate analyses for sex, proportion of front-seat passengers, age, d-BP, BT, GCS, and AIS scores of the neck and abdomen. These variables were included in the logistic regression analysis. Ultimately, male sex (OR: 1.95), front-seat passenger (OR: 1.99), age (OR: 1.04), d-BP (OR: 0.98), BT (OR: 0.54), GCS (OR: 0.74), and AIS of the neck (OR: 1.85) and abdomen (OR: 1.464) were identified as independent predictors of fatality in Period A (Table 7). In Period B, significant differences were found in univariate analyses for sex, age, GCS, AIS score of the abdomen, and FAST results. These variables were included in the logistic regression analysis. Ultimately, male sex (OR: 2.49), front-seat passenger (OR: 1.99), age (OR: 1.05), GCS (OR: 0.77), AIS of the abdomen (OR: 1.40), and FAST positivity (OR: 2.40) were identified as independent predictors of fatality in Period B (Table 8).

**Table 7:** Results of multivariate analyses to identify the factors associated with fatality in Period A (2004–2008).

	Period A (n = 4,684)				
		OR	Lower 95% CI	Upper 95% CI	P-value
Gender	Period A	Ref.			
	**Male**	1.948	1.143	3.321	0.014
Seating position	**Driver**	Ref.			
	**Front seat passenger**	1.985	1.089	3.620	0.025
Age		1.036	1.024	1.047	<0.001
d-BP		0.976	0.960	0.991	0.002
BT		0.539	0.426	0.681	<0.001
GCS		0.740	0.696	0.788	<0.001
AIS of the neck		1.850	1.010	3.389	0.046
AIS of the abdomen		1.464	1.239	1.731	<0.001

d-BP: diastolic blood pressure, BT: body temperature, GCS: Glasgow coma score, AIS: Abbreviated Injury Scale,

OR: odds ratio, 95% CI: 95% confidence interval.

**Table 8:** Results of the multivariate analyses to identify the factors associated with fatality in Period B (2016–2017).

		(n = 3,690)			
		OR	Lower 95% CI	Upper 95% CI	P-value
Gender	Female	Ref.			
	Male	2.493	1.236	5.031	P < 0.001
FAST	Negative	Ref.			
	Positive	2.395	1.204	4.765	P = 0.013
Age		1.050	1.033	1.068	P < 0.001
GCS		0.768	0.707	0.834	P < 0.001
AIS of abdomen		1.401	1.145	1.713	P = 0.001

FAST: focused assessment with sonography for trauma, GCS: Glasgow coma score, AIS: Abbreviated Injury Scale,

OR: odds ratio, 95% CI: 95% confidence interval.

## Discussion

Comparing the two study periods, there was a dramatic increase in the mean age of patients with MVC-related injuries from 45 years in Period A to 53 years in Period B. This trend likely reflects the aging population in Japan, with those aged 65 years or older increasing from 20.8% of the population in 2006 to 27.3% of the population in 2016^22,23^. As the average life expectancy of Japanese women (87.1 years) was higher than that of men (81.0 years) in 2016, the proportion of males decreased from 69.0% to 61.6%^24^.

Comparisons between the two study periods also showed a significant decrease in fatalities from 7.9% in Period A to 4.3% in Period B. Furthermore, the injury severity in most body regions significantly decreased, and other physiological parameters improved significantly. These trends are similar to those found in previous retrospective trauma studies^25–29^. Other studies performed using the same data-bank found that in-hospital mortality decreased over time^27,30^. Because the number of serious or fatal injuries decreased, the present study found that the GCS score of the patients significantly improved, and that the FAST positivity rate significantly decreased.

It is difficult to explain reductions in in-hospital mortality with any single factor. The decrease in the MVC-related fatality rate over time may have been affected by many factors, such as education, law enforcement, and improvement of prehospital and trauma care (including the shortening of the duration between the emergency call and hospital arrival). Education on standard trauma care, such as Japan Prehospital Trauma Evaluation and Care, and Advanced Trauma Operative Management, has been widely promoted^27^. In 2002, the Japanese Association for the Surgery of Trauma published the Japan Advanced Trauma Evaluation and Care (JATEC), which provides doctors with medical treatment guidance for off-the-job training^31^. These educational approaches might contribute to decreased vehicle passenger fatality rates. A previous study in Japan revealed that helicopter transportation and usage of whole-body computed tomography (CT) were associated with improved mortality rates for severe trauma patients^32–35^. Japan has the highest number of CT scanners per capita in the world^36^. Furthermore, in most emergency departments and critical care centres, high-speed CT scanners are located close to the trauma bay. Hybrid emergency rooms, which have recently been installed in several facilities, are also associated with increased survival after potentially fatal trauma^37^.

In the years between the two study periods, laws requiring seatbelt usage in rear seats and strengthened penalties for drunk driving were established. In 2008, the Road Traffic Law was revised so that rear-seat passengers were required to wear a seatbelt, in addition to four-wheeled vehicle drivers and front-seat passengers. The rates of seatbelt use by rear-seat passengers dramatically increased from 8.8% in 2007 to 30.8% in 2008^38^. Because of improved awareness that seatbelts should be fastened while driving, the respective rates of seatbelt use for drivers, front-seat passengers, and rear-seat passengers increased from 93.8%, 83.4%, and 7.5% in 2006 to 98.5%, 94.9%, and 36.0% in 2016^39^.

Following increased public awareness that ‘drunk driving is unacceptable’, police implemented the revised Traffic Law in 2007, which includes stricter regulations and penalties targeting drunk driving and any environments that enable drunk driving. When a person is arrested for drunk driving, an in-depth investigation is conducted not only of the driver, but also of the other vehicle passengers or persons who provided the alcohol to the driver. The revised Road Traffic Law also promotes penalties to fellow passengers or other persons who enabled the drunk driving. After the implementation of this revised Road Traffic Law, the number of traffic collisions caused by drunk driving decreased from 11,625 in 2006 to 3,757 in 2016^40^. We expect further improvements with increased public awareness and decreased social tolerance for alcohol-impaired driving. Furthermore, improved motor vehicle engineering and safety devices might positively affect in-hospital mortality.

The factors independently influencing the occurrence of fatalities in each period were determined by multivariate analyses. From 2004–2008, fatalities were more likely to involve older male front-seat passengers with low d-BP, BT, and GCS values, and high AIS of the neck and abdomen. However, in 2016–2017, being a front-seat passenger or having low d-BP, low BT, and high AIS of the neck were not significant independent predictors of fatality. As mortality rates decreased in the 10 years specified above, the indices related to severe or fatal injuries, such as d-BP, BT, and AIS score of the neck, were not selected. With the introduction of laws on mandating seatbelt use in the rear seat and increased awareness of the importance of seatbelt use, the seatbelt use rate gradually improved, even in the front seats. For front-seat passengers, seatbelt use on general roads increased from 83.4% in 2006 to 94.9% in 2016^41^. Because most people sitting in the front passenger seat are restrained and have airbag protection, being a passenger in the front seat was not significantly associated with fatality in 2016–2017.

In both groups, the associations of low GCS and increased age with an increased fatality rate are well understood. The GCS is well correlated with fatality after head injuries. A systematic review and meta-analysis of trauma patients reported that older patients had higher mortality than younger patients^42,43^. Older patients have higher mortality and morbidity than younger patients, even in MVCs of equal severity^44,45^. It has been suggested that the physical vulnerability of older patients leads to a worse prognosis after MVC^30^. Injury severity after a MVC is influenced by many age-related physical changes, such as muscle weakness, osteoporosis, and reduction in organ system function.

In addition to the AIS of the abdomen, a positive FAST finding became an independent factor influencing fatality in 2016–2017. As the prevalence of seatbelt use increased and airbags became the norm in the driver and front passenger seats, the occurrence of severe chest and head injuries decreased. Seatbelt use prevents severe contact between the chest and abdomen and the vehicle interiors, but the seatbelt itself can apply force that injures the abdominal organs, as the abdomen has no skeletal protection.

With appropriate seatbelt use, the forward movement of the passenger is suppressed during a collision. However, if the seatbelt is not appropriately positioned, there is a higher possibility of abdominal injury caused by the seatbelt^46,47^. A recent study suggested that 40% of rear-seat passengers showed inappropriate seatbelt positioning, with the lap belt moving from the right or left anterior superior iliac spine^48^. This phenomenon may lead to compression of the abdomen, causing abdominal organ injury during a collision. Therefore, abdominal injuries may influence on the outcome. A positive FAST result indicates that abdominal injuries are present. With the increased popularity of the JATEC, FAST quickly became widely performed throughout Japan, providing an additional factor for evaluation of the abdomen.

One limitation of this study is that it did not include all injured vehicle passengers in Japan. However, the JTDB represents trauma cases to a similar degree as databases in North America, Europe, and Oceania. In addition, almost all certificated trauma educational institutions and many critical care centres participated in this database. Because the JTDB is the only prospective, nationwide, hospital-based trauma registry, we believe our analyses provided representative results. Second, information on crashes, such as collision details (a type of car, collision direction, and velocity), seatbelt use, and airbag deployment was not present in the JTDB registry. Because our study was based on medical data, analyses lacking these details do not have decreased reliability. However, future studies should examine connections between accident data collected by the police department and medical data based on hospital records in Japan. Third, the JTDB contained flawed information. In the JTDB, numerical values or applicable items are registered via the website, while freely written information cannot be entered. Therefore, some of the items had missing data. However, as unclear or missing data were excluded before analysis in the present study, the analyzed data had a high level of reliability. Fourth, as this database was hospital-based, cases of instant death pronounced at the collision scene were not included. Furthermore, patients experiencing cardiopulmonary arrest on arrival were excluded from this study. In these cases, the physiological parameters and details of the injuries were not obtained because of the lack of examinations. Therefore, including these out-of-hospital deaths likely would not improve the reliability of the present analyses.

## Conclusion

Our study identified independent factors influencing vehicle passenger fatalities. With the increasing average age in Japan, changing manners of injury in motor vehicle accidents, and development of new traffic safety measures, analyses similar to the present study should be performed regularly in the future.
